# A new kind of polystyrene/polyethyleneimine nanofibres coordinated with palladium for fast and efficient extraction of methotrexate and its polyglutamate metabolites in different matrices†

**DOI:** 10.1039/d5ra00930h

**Published:** 2025-05-08

**Authors:** Li Xie, Jingyi Shen, Qing Shu, Weihong Ge, Xiuhua Yang, Ahad Hussian, Xuejun Kang

**Affiliations:** a College of Animal Science and Technology, Jinling Institute of Technology Nanjing 210038 China; b Department of Pharmacy, Nanjing Drum Tower Hospital Nanjing China; c Nanjing Rubisi Biotechnology Co., LTD Nanjing 211135 China; d Key Laboratory of Child Development and Learning Science (Ministry of Education), School of Biological Science & Medical Engineering, Southeast University Nanjing 210096 China xjkang64@163.com

## Abstract

Methotrexate (MTX) is a drug that has long been used in high doses as an anti-cancer drug and lately in low doses as a treatment for autoimmune diseases. It is necessary to be determined in various matrices because the drug has a narrow therapeutic range and a high persistence in the environment. Since MTX and its polyglutamate metabolites (MTXPGs) have strong polarity and the potential to be converted during sample processing, rapid and efficient extraction of these targets has provided technical challenges for development of analytical methods for them. A new Pd(ii)/polyethyleneimine (PEI)/polystyrene (PS) nanofibre was prepared and characterized by X-ray diffraction, FTIR spectroscopy, thermal analysis, scanning electron microscopy and transmission electron microscopy, *etc.* The nanofibre was applied as a sorbent to extract MTX and MTXPGs in whole blood and MTX in urine and water. The extracted analytes were then desorbed by a water solution containing 10% methanol and 20% ammonium hydroxide (v/v) and eventually quantified by high performance liquid chromatography. The peak area of the target substances in the extraction solution and its concentration were linear in the range of 20.0 (∼37.8 to 1000 ng/mL); intraday and interday RSD were 4.6–6.4% and 5.7–14.6%, respectively. The detection limit of this method was 6.0–11.3 ng/mL. The results showed that the method can be used for the determination of MTX and MTXPGs in human whole blood and MTX in urine/water samples.

## Abstract 2

Conceptual framework of the current study.

## Graphical abstract

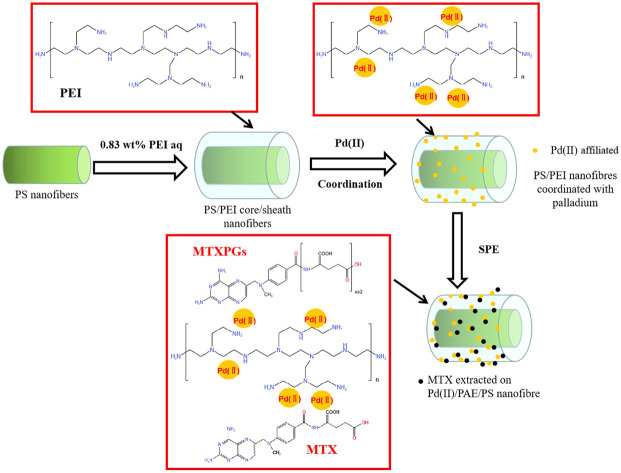

## 1 Introduction

Methotrexate (MTX) is the most frequently utilised anticancer drug in clinical practice, with approximately 80–90% of it being excreted unchanged into aquatic environments.^1^ Large doses of MTX are widely used in clinical therapy of osteosarcosis, acute lymphoblastic leukemia, and malignant lymphoma, and used in small doses to treat rheumatoid joints inflammation and inflammatory bowel diseases. High therapeutic concentrations of MTX might lead to hepatic and pulmonary anomaly and myelosuppression. However, individual differences in MTX are large, and its adverse reactions are frequently reported.^2^ The biotransformation of methotrexate to polyglutamates (*n* = 2–7) (MTXPGs) in normal or cancer cells is mediated by the folylpolyglutamate synthase enzyme. These MTXPGs also exhibit enhanced inhibitory effects on enzymes involved in folate metabolism, thereby playing a crucial role in determining the duration of MTX's action.^3,4^ Since MTXPGs do not easily penetrate the cell membrane and exist in the cell for a long time, some scholars believe that MTXPGs are the true pharmacodynamic substance of MTX and the root cause of the differences between individual.^5^ It is necessary to use total concentrations of MTX and MTXPGs as a key surrogate for clinical drug monitoring. In addition, due to its environmental toxicity, identifying trace amounts of MTX in environmental samples is also needed.

Sample preparation mainly focuses on two points: (i) separation of the matrix components that can interfere with the target molecules analysis and (ii) increase of the target molecule concentration until detectable level into a lower volume,^6,7^ which is often the most time-consuming and labour-intensive step in the analytical procedure, and is critical for the final measurement error.^8^ Currently, a growing interest has been turned to innovative techniques that allow a reduction in sample handling while maintaining high analytical performance.^8^ Greater attention is given to the materials used, to renewable sources, to the reduction of the environmental impact and to the increase of the safety and health of the operators, working through the reduction of volumes of solvents and samples used.^6–8^ However, challenges for the determination of MTX and MTXPGs often arise when analyzing samples due to matrix effects (such as blood/urine/polluted water *etc.*).^9^ Therefore, selecting the appropriate technique for sample preparation is a widely discussed issue when developing analytical methods for measuring MTX in various samples, particularly in the field of therapeutic drug monitoring.^7^

MTX molecule consist of a polyelectrolyte with two carboxyl groups and several nitrogen-containing functional groups.^10^ Since MTX and MTXPGs are very polar, they are almost insoluble in all organic solvents that are miscible with water. Therefore, conventional liquid–liquid extraction method is not suitable for the extraction of MTX and MTXPGs. SPE necessitates high selectivity for the adsorbent, which has been extensively employed by various researchers.^9^ However, previous studies indicated that commercial SPE materials, such as C18 SPE columns, resulted in poor retention of higher-order polyglutamate metabolites, although reasonable for methotrexate, and the application of strong anion exchange SPE columns produced variable and incomplete recoveries for all analytes.^11^ OASIS HLB solid phase extraction (Waters) column could purify MTX and some of MTX metabolites, but the recovery rate of MTX reported only 39%.^12^ Some researchers focused on the preparation of new adsorbents to solve the problem of pretreatment of these target compounds. Liu *et al.* utilised molecularly imprinted polymers (MIPs) as artificial recognition materials for extracting and then determining of MTX in human serum by High Performance Liquid Chromatography (HPLC).^13^ Hansen *et al.* developed an electromembrane extraction method for the simultaneous determination of MTX and its metabolites, and the recoveries of 23–53% were achieved.^14^

Nitrogen-containing polymers, such as polyethyleneimine (PEI), poly(4-vinylpyridine) (PVP), polyaniline (PANI), and poly(Schiff bases) are able to efficiently chelate metal ions such as Pd(ii) or Cu(ii) *via* its multidentate donor ligand with nitrogen-binding sites.^15–18^ These reactions lead to the formation of complexes in which nitrogen atoms coordinate with palladium ions.^19^ Based on these, the paper proposed a kind of nanofibres feature Pd(ii) coordination on the surface of a layer of PEI polymer coated on the polystyrene (PS) nanofibres. The newly prepared nanofibres were used as solid phase extraction (SPE) material to extract MTX and MTXPGs from samples which had not been explored by other researchers. The proposed Pd(ii)/polyethyleneimine/polystyrene (Pd(ii)/PEI/PS) nanofibres is the first time utilising Pd(ii) loading on polymers as an SPE adsorption materials, which can significantly improve the extraction efficiency of highly polar MTX and MTXPGs on nanofibres.

## 2 Experimental

### 2.1. Chemicals, reagents, and samples

All chemicals and reagents used in the study were of analytical grade. Ammonium salts of MTX, MTXPG1, MTXPG2, MTXPG3 were purchased from Schircks Laboratories (Jona, Switzerland). Polystyrene (PS, retained molecular weight: 18 500 Da) and polyethyleneimine (PEI) were purchased from Aladdin Company (China). The Sep-Pak silica cartridges used for sample treatment was purchased from Waters (Milford, MA, USA).

Standard stock solution was prepared with 0.1 mg/mL of MTX and MTXPGs in pure water and stored at 4 °C in a brown flask. The working solutions were prepared as appropriate dilutions of the stock solutions.

Blank human urine and whole blood were obtained from non-treated unidentified volunteers. Urine was collected into aseptic urine cups and then transferred into polypropylene tubes and immediately stored in a −20 °C freezer.

### 2.2. Chromatography method

The HPLC system consisted of a SHIMADZU LC-20AD high performance liquid chromatography (Shimadzu Corporation, Japan) and a SPD-10AD UV detector (Shimadzu Corporation, Japan). Chromatographic separation was operated on the HPLC system with an VP-ODS Shimadzu C18 (5 μm, 250 mm × 4.6 mm) cartridge. The targets were separated in an isocratic elution with a binary mobile phase consisting of 5% acetonitrile (v/v), 1.6% Na_2_HPO_4_ (w/v), and 0.7% citric acid (w/v). The flow rate was kept constant at 1.0 mL/min. The injection volume for the samples was set at 20 μL, and the UV detection wave length was 302 nm.

### 2.3. Characterization

The phase composition of sample was characterized by X-ray diffraction (XRD, Haoyuan DX-2700BH, China) with Pd Lα radiation at 30 mA and 40 kV. The 2*θ* range, step sampling time and step angle are 0–80°, 0.2 s and 0.02°, respectively. The morphology and microstructure of the samples were observed by emission scanning electron microscope (SEM, Zeiss Sigma 300, Germany) equipped with energy-dispersive spectroscopy (EDS). Transmission electron microscopy (TEM) images were carried out on a JEM-2010 microscope (Japan), and the parameters of specific surface and porosity were analysed using Micromeritics ASAP 2020 (USA). Thermogravimetric analysis (TGA) was carried out on Shimadzu DTG-60 thermogravimetric analyser with a ramping rate of 10 °C/min in 50 mL/min of air flow. Fourier transform infrared (FITR) spectra were recorded on a Thermo Nicolet Magna 550 spectrometer. The surface electronic states were analysed by X-ray photoelectron spectroscopy (XPS, PerkinElmer PHI 5000C ESCA). All the binding energy values were calibrated using C_1s_ = 284.6 eV as a reference. The SPE columns and air-pumped array SPE processors were purchased from Dongqi Bio-technology Co., Ltd (Suzhou, China).

### 2.4. Preparation of Pd(ii)/PEI/PS nanofibres

The PS nanofibres were prepared as described in the literature.^20^ The PEI was dissolved into water to form a 30 wt% of polymer aqueous solution, and the solution was diluted with a mixed solvent (water : ethanol = 1 : 2, w/w) to 0.83 wt%. Next, the PS nanofibres were immersed in the freshly prepared aqueous solution of 0.83 wt% PEI at room temperature for 6.0 h. After the incubation, nanofibres were washed with deionised water several times and dried overnight in an oven at 40 °C to get polyethyleneimine/polystyrene (PEI/PS) nanofibres.

The palladium chloride was dissolved in 0.1 M HCl solution with a certain concentration. The PEI/PS nanofibres were immersed in the 0.1 M HCl palladium chloride solution for at least 2 h. Then the nanofibres were washed with deionised water several times and dried overnight in an oven at 40 °C to get Pd(ii)/PEI/PS nanofibres. The whole preparation procedure of Pd(ii)/PEI/PS nanofibres is schematically shown in a graphical abstract.

### 2.5. Extraction of MTX and MTXPGs from samples

Samples were allowed to thaw at room temperature. Prior to separation, the samples were centrifuged (4000 rpm, 4 min.) to remove the suspended particles and stored at 4 °C until the solid-phase extraction (SPE) separation step. The extraction was operated using an air-pumped semiautomatic arrayed SPE processor. The pictures of the SPE columns and the SPE processor are shown in Fig. S1.† The SPE columns were packed with Pd(ii)/PEI/PS nanofibres (5 mg for each column) as the sorbents at the tip end. For the extraction, aliquots of 0.2–5 mL sample was added into each SPE column, and then the sample was pushed out of the columns using an air-pressure processor connected to a vacuum pump. Compounds were adsorbed by the Pd(ii)/PEI/PS nanofibres when the sample solution flowed through. Then, 100 μL of water solution containing 10% methanol and 20% ammonia water (v/v) was used to elute the targets from the Pd(ii)/PEI/PS nanofibres. The elution was gathered and vortexed for further determination.

For aqueous samples such as water or urine, the loading volume was 0.5 mL to 5 mL, while for viscous and sparse samples such as whole blood, a protein precipitation step using silver nitrate solution was used. After protein precipitation, the samples were centrifuged (12 000 rpm, 5 min) and 200 μL of supernatant was transferred to SPE as previously mentioned.

### 2.6. Static adsorption studies

Pd(ii)/PEI/PS nanofibres (10 mg) were put in the cuvette containing 10 mL of the solution (100 μg/mL of each analyte) under constant temperature of 25 °C. Then target compound in the liquid phase was determined at time intervals of 1, 3, 5, 10, 15, 20, 25, 30 and 60 min. The adsorption capacity (*q*_*t*,_ mg/g) of each compound was calculated by the equation:^21^*q*_*t*_ = (*C*_0_ − *C*_*t*_) × (*V*/*M*)where *C*_0_ and *C*_*t*_ are the concentrations of each analyte at initial and at time *t*, respectively; *V* is the volume of solution; *M* is the weight of Pd(ii)/PEI/PS.

The sorption isotherm experiments were conducted in 2 mL working solutions with initial concentration of 0.5, 5, 7.5, 20, 35, 50, 62.5, 70 and 90 μg/mL of each analyte at room temperature. The concentration of the solutions was measured after 2 h of sufficient adsorption equilibrium. The equilibrium adsorption capacity (*q*_e_, mg/g) of each analyte was calculated by the following equation.^21^*q*_e_ = (*C*_0_ − *C*_e_) × (*V*/*M*)where *C*_e_ is the equilibrium concentration.

The equilibrium data under the three temperatures were simulated using two well-known isotherm models, Langmuir model and Freundlich model.^22^

## 3 Results and discussions

### 3.1. Characterisation

#### 3.1.1 Material optimization and morphological characterization

**Fig. 1:**
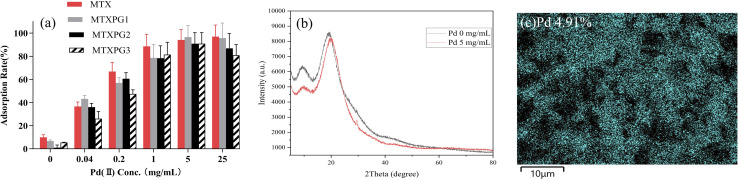
Material optimization and characterization of Pd elements: (a) the adsorption rate of MTX and MTXPGs in water samples by using Pd/PEI/PS nanofibres prepared with 5 mg/mL Pd solution; (b) XRD pattern of PEI/PS nanofibres and Pd/PEI/PS nanofibres prepared with 5 mg/mL Pd solution; (c) EDS-mapping graphs of Pd/PEI/PS nanofibres prepared with 5 mg/mL Pd solution.

Pd(ii)/PEI/PS nanofibers were prepared with different concentrations of palladium chloride (0, 0.04, 0.2, 1, 5, 25 mg/mL). The adsorption rates of the fibers with different palladium chloride contents for four target substances (500 ng/mL) were investigated. As shown in Fig. 1a, the adsorption rate was gradually increased with the increase of palladium chloride content in the fibers until the palladium chloride content reached 5 mg/mL. Therefore, the Pd(ii)/PEI/PS nanofiber prepared with 5 mg/mL of palladium chloride was used as the preferred adsorbent. The optimized nanofibers' EDS-mapping graph is shown in Fig. 1b, and it is shown that Pd element distributed uniformly in the nanofibers. Fig. 1c shows the XRD pattern of the optimized nanofibres. At 2*θ* degree about 20°, the PEI/PS nanofibres exhibit a distinct broad and diffuse PEI diffraction peak, reflecting the success of PEI modification and its amorphous structure.^23^ The XRD diffraction peaks of Pd/PEI/PS nanofibres are similar to those of PEI/PS, but no diffraction peaks corresponding to the Pd element are observed. However, the diffraction peak intensity of Pd/PEI/PS is significantly lower than that of PEI/PS. This might be because palladium exists in the amorphous state or in a highly dispersed form on the material surface, so no obvious characteristic peaks are shown in the XRD spectrum.^24^ However, Pd modification significantly reduces the crystallinity of PEI/PS, which interferes with the crystallinity of PEI/PS, indicating Pd(ii) coordination on the surface of PEI polymer.

**Fig. 2:**
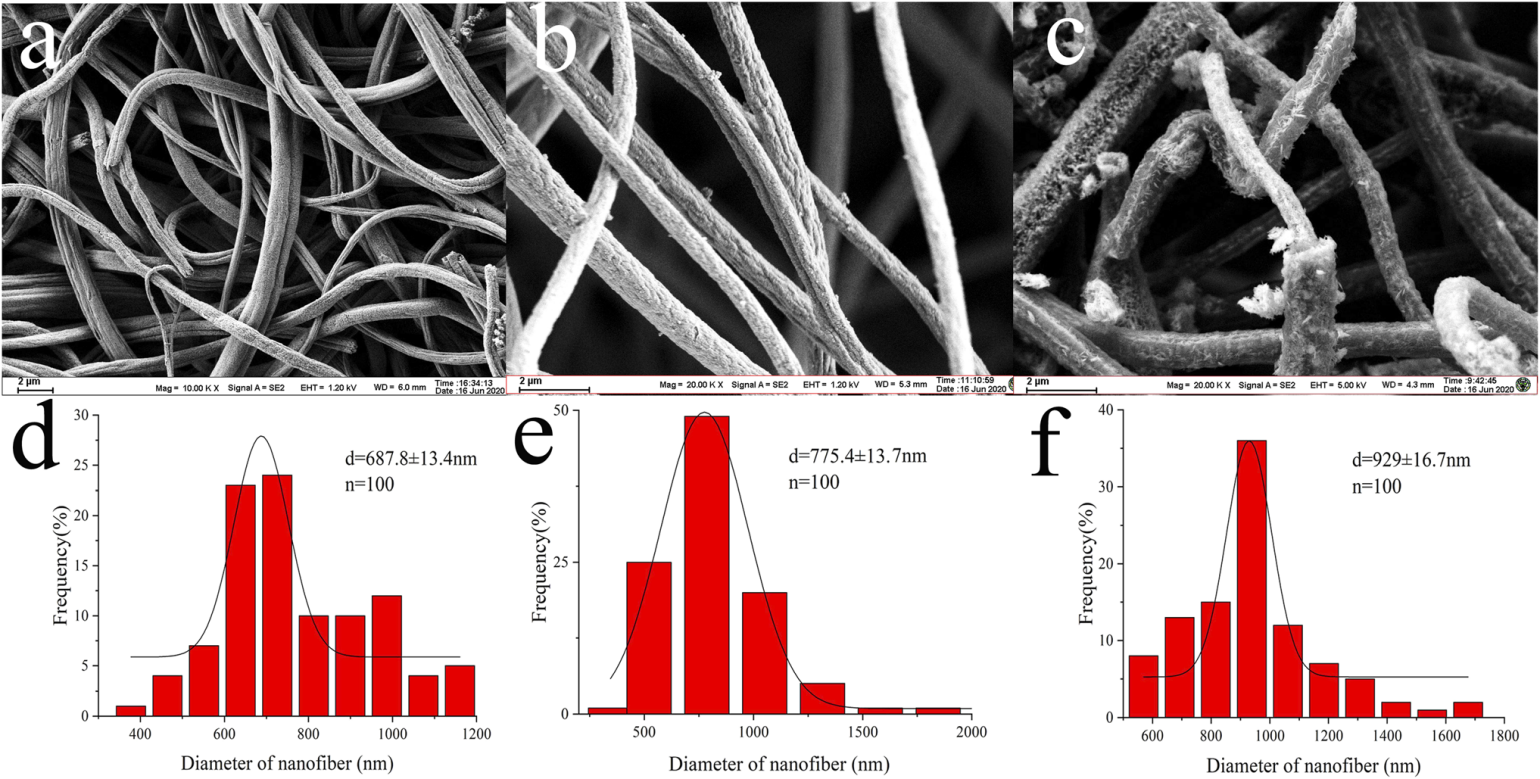
SEM images of (a) PS electrospun nanofibres, (b) PdCl_2_ coating PS nanofibres, and (c) Pd(ii)/PEI/PS nanofibres, and the size distribution histogram of (d) PS nanofibres, (e) the PdCl_2_ coating PS nanofibres, and (f) Pd(ii)/PEI/PS nanofibres.

**Fig. 3:**
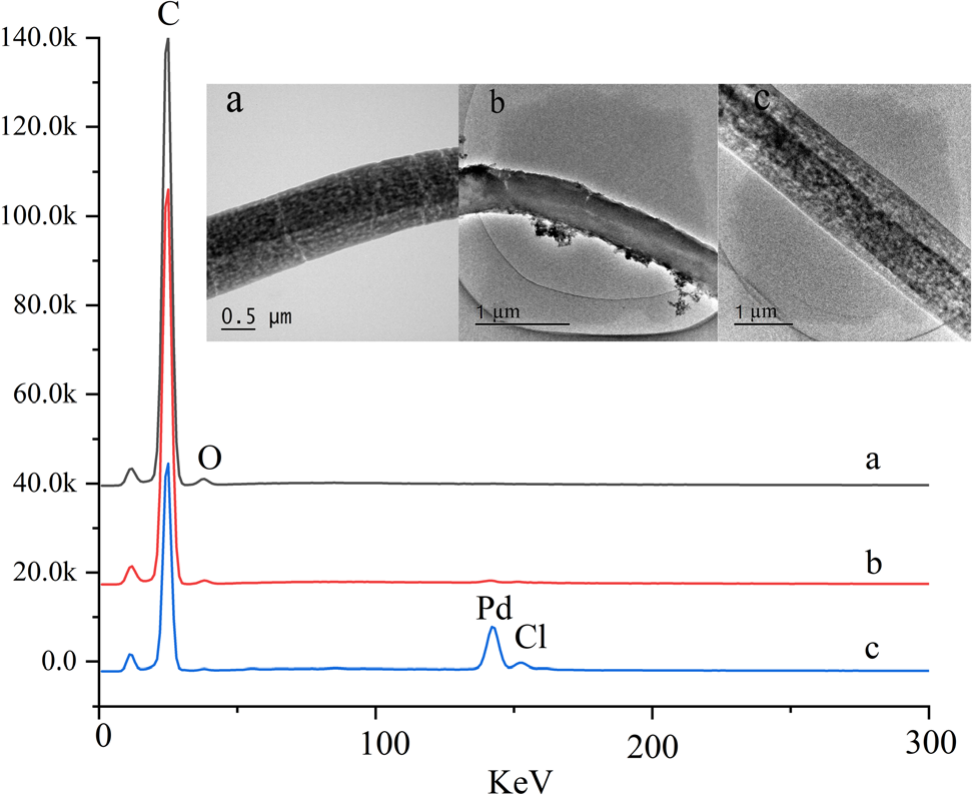
TEM images and EDX spectra of (a) PS electrospun nanofibres, (b) the PdCl_2_ coating PS nanofibres on and (c) Pd(ii)/PEI/PS nanofibres.

To investigate the extraction mechanism of the Pd(ii)/PEI/PS nanofibres for MTX and MTXPGs, PdCl_2_ coated PS nanofibres were prepared for comparison by immersing PS nanofibres in the PdCl_2_ solution for several hours, followed by washing and direct drying. The SEM images and diameter distribution are presented in Fig. 2b and e, respectively. As depicted in Fig. 2a and d, PS nanofibres were generated with a mean diameter of 654 ± 30.7 nm. However, the chemical assembly of PEI and PdCl_2_ resulted in an increase in the diameter of the nanofibres to 929 ± 16.7 nm (Fig. 2c and f). The formation of a core/sheath structure in Pd(ii)/PEI/PS nanofibres can be clearly observed (Fig. 3c). The increase in nanofibre diameter can be attributed to the swelling of the PEI layer. In Fig. 3b, a randomly distributed agglomerated layer was observed on the surface of the fibres, and the fibres exhibited a diameter distribution similar to that of PS nanofibres.

The modification of PEI and PdCl_2_ onto the PS nanofibres was characterised using transmission electron microscopy (TEM) imaging and energy dispersive X-ray spectroscopy (EDX). A clearly observed agglomerated layer was present on the surface of the PdCl_2_ coated PS nanofibres, and its EDX spectrum showed no detectable Pd elements, indicating the unsuccessful assembly of PdCl_2_ along the nanofibre surface (Fig. 3b). In contrast, the Pd(ii)/PEI/PS nanofibres exhibited a two-tier structure, with Pd elements clearly visible in the EDX spectrum (Fig. 3c). And the FTIR spectroscopy, X-ray photoelectron spectra analysis and thermal analysis of the materials were demonstrated in ESI (Fig. 1–3),† which indicate that Pd(ii) ions were coordinated with PEI and the MTX coordinate with Pd on the Pd(ii)/PEI/PS nanofibres.

#### 3.1.2 Surface and porosity analysis

The textural properties of the nanofibres are presented in Table 1, including the surface areas calculated using the BET equation, as well as the pore volumes and pore sizes of the three tested nanomaterials corresponding to Fig. 2 and 3. It is evident that the BET surface area and pore size of Pd(ii)/PEI/PS nanofibres were found to be decreased compared to PS, while the pore volume was greatly enhanced. In contrast, the PdCl_2_-coated PS nanofibres hardly contained any surface pores.

**Table 1:** Textural properties of the PS nanofibres, PdCl_2_ coating PS nanofibres, and Pd(ii)/PEI/PS nanofibres^a^

Material	BET surface area (m^2^/g)	Pore volume (cm^3^/g)	Pore size (nm)
PS nanofibres	18.99	0.27	53.63
PdCl_2_ coating PS nanofibres	0.83	0.027	ND
Pd(ii)/PEI/PS nanofibers	12.23	8.33	27.15

a ND: not detected.

### 3.2. The effect of salt concentration and pH on the extraction of MTX and MTXPGs

**Fig. 4:**
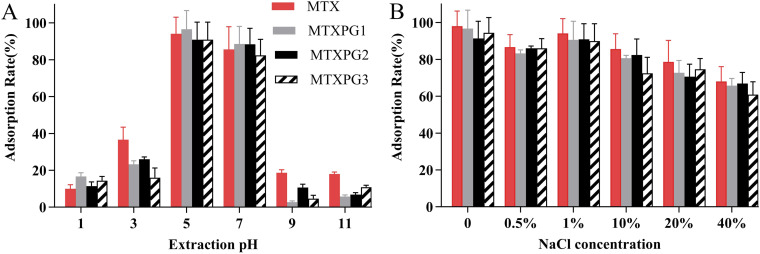
Extraction condition optimizations for (A) the extraction pH and (B) the influence of the concentration of salt (NaCl) on the extraction.

The extraction pH and the influence of salts on the adsorption rate were evaluated (adsorption rate = (spiked amount − post-column amount)/(spiked amount) × 100%). The pH was evaluated by adjusting the pH of spiked water samples (100 ng/mL) from 1–11. The effect of salt concentration on extraction was evaluated by the addition of NaCl at different concentration levels from 0 to 40% (w/v). As shown in Fig. 4A, the optimal extraction pH was determined to be 5–7 for MTX and MTXPGs. As depicted in Fig. 4B, the addition of salt had a slight negative impact on the adsorption rate. This could be due to an increase in aqueous solution viscosity caused by the addition of salt, resulting in hindered mass transfer and reduced adsorption rate. Furthermore, the addition of salt would diminish the interaction between the targets and the sorbent surface, thereby reducing the adsorption rate. Therefore, minimizing the addition of salt is conducive to improving the extraction efficiency.

**Fig. 5:**
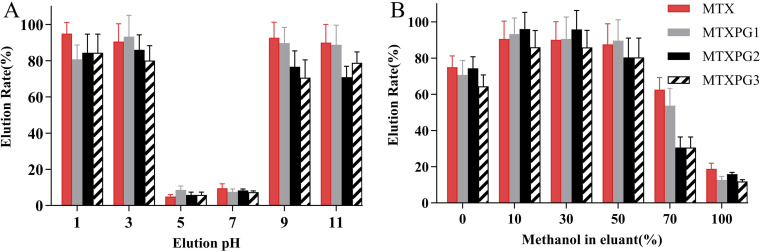
Optimization of the eluant for (A) the eluant pH and (B) the ratio of methanol used in the eluant.

The influence of pH and the concentration of methanol on the elution rate were evaluated (elution rate = eluted amount/spiked amount × 100%). Fig. 5A demonstrated that when the pH of the elution solution became either alkaline or highly acidic, the targets were eluted from the adsorbent. Furthermore, methotrexate was unstable in strongly acidic solutions, Fig. 5B optimized using 10% methanol for elution. Therefore, the eluent was consist of 10% methanol with a pH adjusted to 9–11.

### 3.3. Extraction performance of the Pd(ii)/PEI/PS nanofibres for environmental and biological samples

#### 3.3.1 Extraction of MTX from environmental samples

The presence of refractory-to-degradation MTX in wastewater has been identified (1.6 to 4756 ng/L).^25^ Due to the complex matrix and low compound concentrations, pretreatment of these samples was an essential step. Most of the studies utilised solid-phase extraction (SPE) for sample preparation,^26^ employing various commercial cartridges with different extraction efficiencies. It should be noted that MTXPGs only exist intracellular, formed by catalyzing MTX through glutamine synthetase in the cytoplasm. The long-chain structures of MTXPGs are hydrolyzed by γ-glutamyl hydrolase present in plasma and re-generated as methotrexate. Therefore, MTXPGs do not exist in plasma, urine or natural water bodies.^27^ The paper used stilled water, whole blood sample to explore MTX and MTXPGs' adsorption rate on sorbents, and urine samples to explore the adsorption rate of MTX only.

**Fig. 6:**
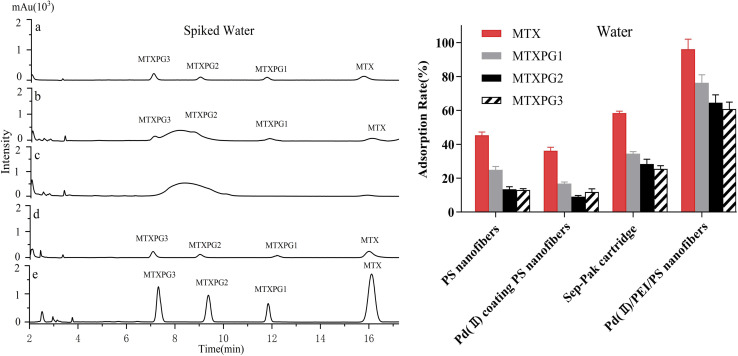
Left: HPLC chromatograms of spiked water (10 ng/mL), (a) standard solution consisting of MTX and MTXPG1-3, (b) SPE using PS nanofibres, (c) SPE using PdCl_2_ coating PS nanofibres, (d) SPE using a conventional Sep-Pak cartridge, and (e) SPE using Pd(ii)/PEI/PS nanofibres. Right: The adsorption rate of MTX and MTXPGs in water samples by using PS nanofibres, PdCl_2_ coating PS nanofibres, conventional Sep-Pak cartridges and Pd(ii)/PEI/PS nanofibres, respectively.

The spiked water (10 ng/mL) was prepared and subjected to extraction using three types of nanofibres (PS nanofibres, PdCl_2_ coated PS nanofibres, and Pd(ii)/PEI/PS nanofibres) as well as a conventional Sep-Pak silica cartridge.^28^ The HPLC chromatograms are shown in Fig. 6 left. And the adsorption rate of these materials was also analyzed as described in Fig. 6 right, which results clearly demonstrate that Pd(ii)/PEI/PS nanofibres exhibited higher adsorption efficiency for the target compounds compared to the PS-based nanofibres and Sep-Pak cartridge. The chromatographic analysis of the spiked water samples revealed that the Pd(ii)/PEI/PS nanofibres exhibited higher adsorption efficiency for the target compounds and the adsorption rate did not decrease significantly after repeated use for 5–8 times.

It is evident that Pd(ii)/PEI/PS nanofibres exhibit superior adsorption efficiency for the target compounds compared to the other two types of nanofibre, while PdCl_2_ coated PS nanofibres, with a small surface area-to-volume ratio, show limited adsorption capability for the targets. The surface characteristics of nanofibres may contribute to their adsorption ability for the target compounds. However, the chemical affinity between Pd(ii) anchored on the fibers and the multiple electronegative elements, such as nitrogen element, in the target molecules may be the decisive factor. Extensive research has been conducted on the chemical behaviour of Pd(ii) complexes, which has significantly advanced the progress in developing new Pd(ii) complexes.^29^ The author thoroughly discussed the reactions of Pd(ii) complexes with various sulphur and nitrogen donor biomolecules under different experimental conditions. The Pd(ii) had been used to complex with biogenic amines,^19^ glycyl-*l*-aspartic acid.^30^ PEI is an aliphatic polyamine that contains primary, secondary, and tertiary amines, making it a suitable polymer for producing polycationic nanofibres.^31^ PEI is water-soluble, which makes it highly suitable for cross-linking with Pd(ii). The recovery of the spiked sample extracted using a Sep-Pak silica cartridge was significantly lower than that of Pd(ii)/PEI/PS nanofibres, confirming the previously deduced interaction. This indicates the successful modification of Pd(ii)/PEI/PS nanofibres with Pd and that the interaction between Pd and electronegative elements facilitated the extraction of MTX and MTXPGs from the samples.

#### 3.3.2 Extraction of MTX/MTXPGs from whole blood and MTX from urine

**Fig. 7:**
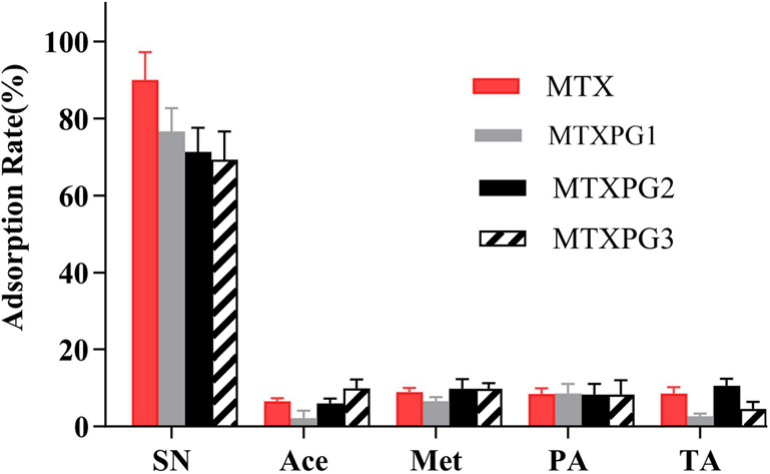
Different precipitation methods for getting rid of the protein in the whole blood sample (SN: silver nitrate; Ace: acetonitrile; Met: methanol; PA: perchloric acid; TA: trichloroacetic acid).

Directly injecting protein-rich biological samples can cause column obstruction and reduce recovery, sensitivity, and specificity in the analysis. Therefore, it is necessary to remove proteins to protect the columns and control matrix effects.^9^ Protein precipitation for whole blood is typically achieved through three methods: precipitation with organic solvent, acid or saline solution. To extract polar compounds from whole blood, researchers have utilised various substances, including acetonitrile, methanol, perchloric acid, trichloroacetic acid,^32^ and silver nitrate.^33^ In order to optimise the protein precipitation method, a series of experiments were conducted as shown in Fig. 7. The results indicated that adding an organic solvent to the sample would result in a significant loss of certain target compounds. Furthermore, the removal of these interfering substance always involved nitrogen drying or redissolving, which further increased the analytes loss. Adjusting the sample pH was also necessary when precipitating with acid prior to subsequent processing. The results demonstrated that 40% silver nitrate solution retained the highest number of analytes in the eluent. The mechanism involves the binding of silver ions to the thiol groups on the protein, forming a silver protein complex. This study demonstrated the satisfactory performance of silver nitrate as a precipitating agent for protein separation from whole blood.

**Fig. 8:**
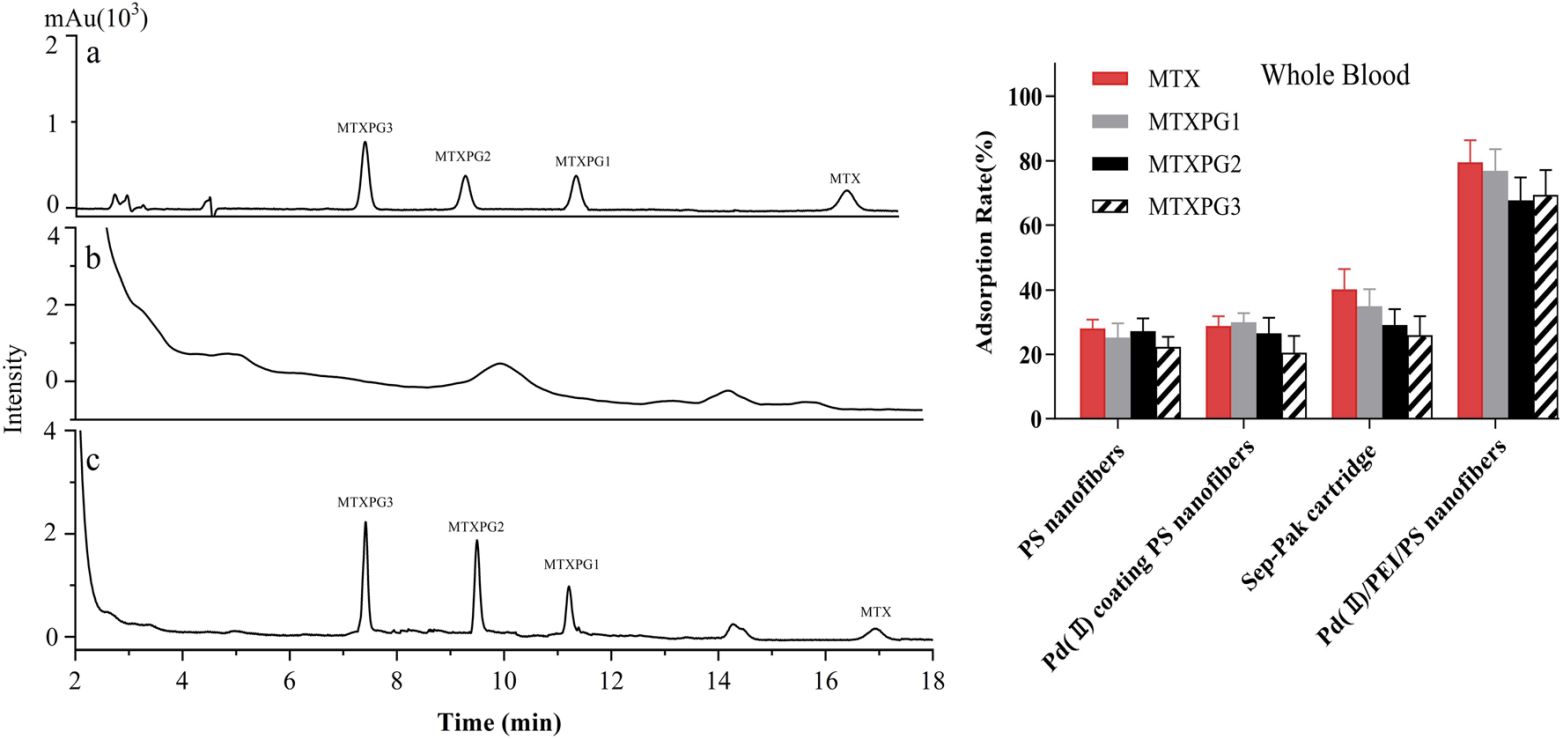
Left: HPLC chromatograms of spiked whole blood samples with MTX and MTXPGs at a concentration of 50 ng/mL (a) standards, (b) treated with conventional Sep-Pak cartridge, (c) treated with SPE using Pd(ii)/PEI/PS nanofibres, Right: The adsorption rate of MTX and MTXPGs in whole blood samples treated by using PS nanofibres, PdCl_2_ coating PS nanofibres, conventional Sep-Pak cartridges and Pd(ii)/PEI/PS nanofibres, respectively.

For comparison, the spiked whole blood samples containing MTX and MTXPGs at a concentration of 50 ng/mL, were extracted with conventional Sep-Pak column and Pd(ii)/PEI/PS nanofibres. The HPLC chromatograms were shown in Fig. 8 left. The extraction efficiencies are listed in Fig. 8 right. It was clearly seen that Pd(ii)/PEI/PS nanofibres exhibited superior adsorption efficiency for the target compounds in whole blood samples. The chromatographic analysis of the spiked whole blood samples revealed that the Pd(ii)/PEI/PS nanofibres exhibited higher efficiency and better purification for the target compounds compared to conventional cartridges.

**Fig. 9:**
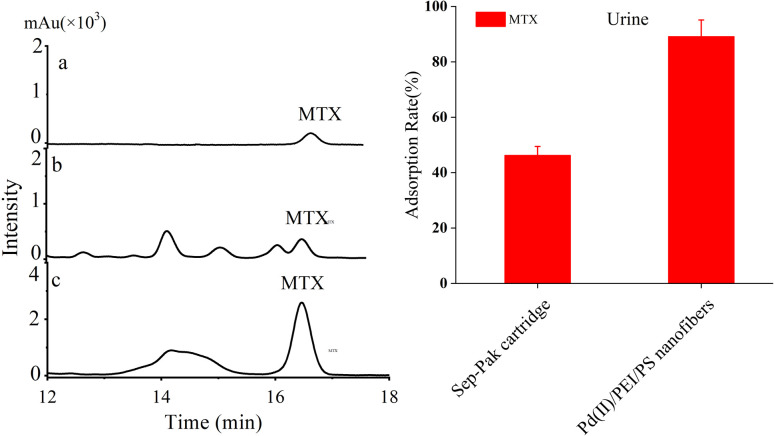
Left: HPLC chromatograms of spiked urine samples with MTX at a concentration of 50 ng/mL (a) standards, (b) treated with conventional Sep-Pak cartridge, (c) treated with SPE using Pd(ii)/PEI/PS nanofibres, Right: The adsorption rate of MTX in urine samples by using conventional Sep-Pak cartridges and Pd(ii)/PEI/PS nanofibres, respectively.

The spiked urine samples containing MTX at a concentration of 50 ng/mL was also extracted. As shown in Fig. 9, Pd(ii)/PEI/PS nanofibres also exhibited superior adsorption efficiency for the target compounds in urine samples.

#### 3.3.3 Static adsorption studies

**Fig. 10:**
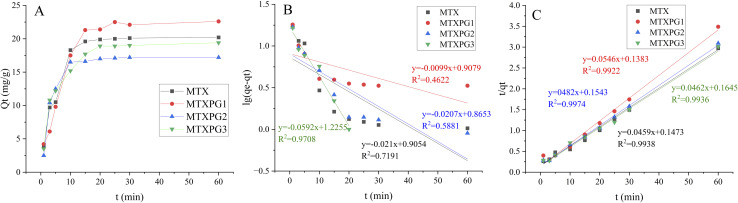
Adsorption kinetic models. (A) Adsorption capacity–time curves at *T* = 298 K, *C*_0_ = 100 mg/L; (B) pseudo-first-order kinetic model; (C) pseudo-second-order kinetic model.

The adsorption capacity–time curves of MTXs on Pd(ii)/PEI/PS nanofibres are shown in Fig. 10A. It was obvious that the adsorption capacity of MTXs increased with adsorption time. All adsorption processes reached equilibrium in a short time. The correlation coefficient (*R*^2^) provided that the pseudo second-order kinetic was better to describe the MTXs adsorption process. The linear form of the pseudo-first-order and the pseudo-second-order models can be expressed as

1 lg(*q*_e_ − *q*_*t*_) = lg *q*_e_ − *k*_1_*t*/2.303

2 *t*/*q*_*t*_ = 1/*k*_2_*q*_e_^2^ + *t*/*q*_e_

where *q*_e_ and *q*_*t*_ are the adsorption capacities at equilibrium and at time *t*, respectively.^34^

**Fig. 11:**
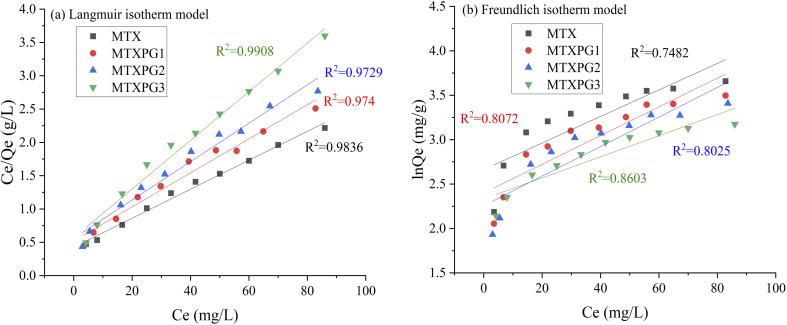
Adsorption isotherms for the adsorption of MTXs on Pd(ii)/PEI/PS nanofibers (a) Langmuir model; (b) Freundlich model.

In order to illustrate the adsorption capacity and equilibrium distribution of MTXs in this study, Langmuir and Freundlich isotherm models were applied to describe the adsorption equilibrium characteristics of MTXs on Pd(ii)/PEI/PS nanofibres, respectively. Eqn (3) and (4) give the linear equations of Langmuir and Freundlich models, respectively.^35^Fig. 11 shows the linear histogram of Langmuir and Freundlich isotherm models. The correlation coefficient between Langmuir and Freundlich model shows that the experimental equilibrium data of MTXs adsorption on Pd(ii)/PEI/PS nanofibres was most consistent with the Langmuir isotherm model (*R*^2^ > 0.97), indicating that the adsorption process is a single-layer process. At the same time, calculated by the Langmuir isotherm model equation, the maximum adsorption capacity of MTXs was 36.4 mg/g, 30.6 mg/g, 26.9 mg/g, and 23.2 mg/g for MTX, MTXPG1, MTXPG2, MTXPG3, respectively.3
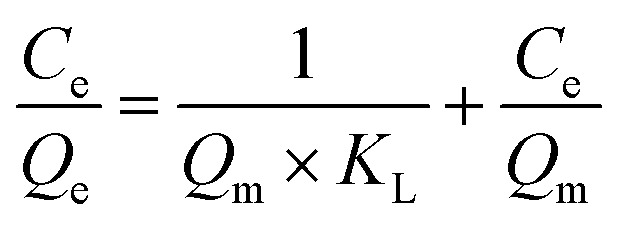
4
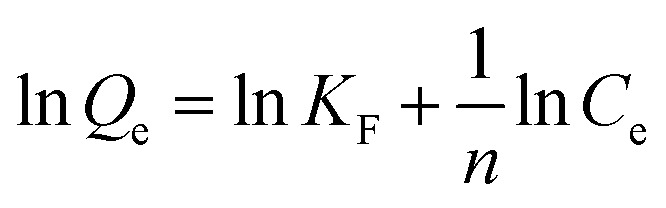
where *C*_e_ (mg/L) is MTXs concentration in equilibrium; *Q*_e_ (mg/g) is the amounts of adsorbed MTXs at equilibrium; *Q*_m_ (mg/g) is the maximum adsorption capacity; *K*_L_ (L/mg) and *K*_F_ (L/g) are the adsorption equilibrium constants for Langmuir and Freundlich models, respectively.

#### 3.3.4 Validation of the method

Quality parameters of the new method were evaluated under optimized conditions for water, blank urine and blank whole blood samples, respectively. The limit of detection (LOD) was obtained at a signal-to-noise ratio of 3 : 1. The limit of quantification (LOQ) was defined as a signal-to-noise ratio of 10 : 1. The linear calibration range, LOD, and LOQ with this new method were calculated, and the results are listed in Table 2. Results showed that the correlation coefficient for targets were all over 0.99. The LOD and LOQ of MTX in urine were 6.00 and 20.00 ng/mL, and in water and whole blood were from 5.00–9.00 and 15.00–36.70, 8.16–11.34 and 33.10–37.80, respectively.

**Table 2:** Analytical parameters for the HPLC method

Analytes	Samples	Linear range (ng/mL)	*R* ^2^	LOD (ng/mL)	LOQ (ng/mL)
MTX	Water	15.00–1000.00	0.999	5.00	15.00
	Urine	20.00–1000.00	0.996	6.00	20.00
	Whole blood	28.80–1000.00	0.990	8.64	28.80
MTXPG1	Water	21.30–1000.00	0.998	7.80	21.30
	Whole blood	33.10–1000.00	0.991	8.16	33.10
MTXPG2	Water	30.20–1000.00	0.995	8.07	30.20
	Whole blood	37.80–1000.00	0.992	11.34	37.80
MTXPG3	Water	36.70–1000.00	0.994	9.00	36.70
	Whole blood	35.2–1000.00	0.998	10.56	35.20

The extraction recoveries of the pharmaceuticals were estimated by spiking each analyte in distilled water at concentrations of 20, 100, and 1000 ng/mL for HPLC-UV (Table 3). The intra-day and inter-day precision and accuracy were evaluated with samples spiked with targets at concentrations of 20, 100, and 1000 ng/mL according to the above pretreatment method for five sequential days. The spiked recovery was estimated as:SR% = (*C*_d_ − *C*_i_) × 100/(*C*_s_)where SR is the spiked recovery (%), *C*_d_ is the detected quantity of targets in the spiked effluent sample, *C*_i_ is the initial amount of target compounds present in the sample, *C*_s_ quantity of targets spiked into the sample.

**Table 3:** Recovery, intra-day and inter-day repeatability expressed as RSD% (*n* = 5)

Analytes	Sample	Spiked conc. (ng/mL)	SR (%)	Intraday (RSD%)	Interday (RSD%)
MTX	Water	20	121.3	11.2	14.8
		100	98.6	9.5	15.2
		1000	100.2	3.8	8.2
	Urine	20	110.1	10.1	15.6
		100	105.2	8.9	13.1
		1000	101.4	4.7	7.4
	Whole blood	20	117.7	11.1	17.6
		100	94.9	10.9	10.1
		1000	102.4	4.6	8.9
MTXPG1	Water	20	121.1	12.1	15.3
		100	93.1	8.5	10.4
		1000	109.2	6.5	6.8
	Whole blood	20	107.6	12.1	16.5
		100	94.6	10.1	10.5
		1000	86.1	7.9	6.1
MTXPG2	Water	20	112.7	16.4	14.6
		100	98.8	10.5	11.1
		1000	94.6	6.9	5.7
	Whole blood	20	108.4	9.1	11.3
		100	87.3	9.5	10.4
		1000	86.0	6.4	8.1
MTXPG3	Water	20	103.1	10.9	12.3
		100	91.2	5.8	8.3
		1000	80.1	6.9	8.1
	Whole blood	20	105.1	11.1	10.6
		100	87.6	10.7	12.4
		1000	76.3	6.7	8.8

The inter- and intra-day precision of relative standard deviation (RSD) for MTX in urine were 4.7–10.1% and 7.4–15.6% (*n* = 5), for the MTX and MTXPGs in water and whole blood were 3.8–16.4% and 5.7–15.6%, 4.6–13.5% and 6.1–17.6% (*n* = 5), respectively.

### 3.4. Comparison with previously reported methods and greenness assessment

As mentioned in the introduction section, according to our knowledge, number of chromatographic methods are reported for the determination of MTX, mainly in plasma, which were based mainly on LC coupled with MS/MS detection,^36^ while in our proposed method, focusing on the evaluation of the new pretreatment of sample, a simple HPLC assay was selected. Table 4 presents a thorough comparison between the proposed method in this study and five other reported methodologies using LC-UV method. According to our knowledge, this is the first study using nanomaterials to decontaminate samples. The other methods pretreated the samples mostly with commercial C18 SPE cartridges^37,38^ or just performed simple centrifugation or protein precipitation steps.^33,39,40^ Compared with other reported methods, the proposed method consumed less organic solvent, saved more pretreatment time, and the detection sensitivity and recovery rate were satisfactory.

**Table 4:** Comparison of the proposed method to chosen reported methodologies using HPLC-UV determination

Sample	Sample preparation	Application	Linearity	LOD	LOQ	Recovery (%)	Organic solvent used (mL)	Preparation time	AGREEprep	AGREE
Proposed method (water, urine, blood)	SPE with PS/PEI/Pd nanofibres	Multiple samples available	25–1000 μg/L	6 μg/L	20 μg/L	94.9–117.7	0.1	4–9 min	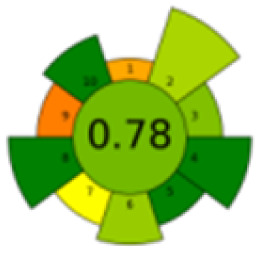	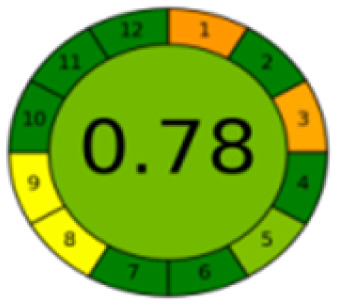
Serum^33^	PP with AgNO_3_ solution	Clinical studies	50–10000 μg/L	6 μg/L	20 μgL	97.5	0.05	>11 min	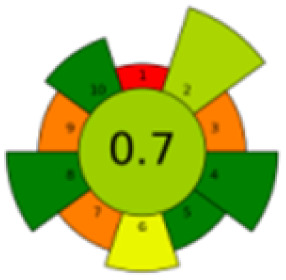	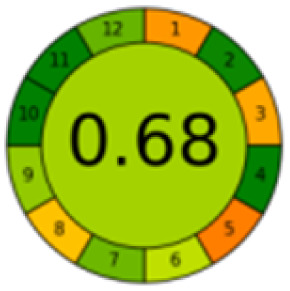
Serum^37^	SPE with Isolute HAX catridge	Clinical studies	155–360 nM	155 nM	—	103 ± 5	14.5	—	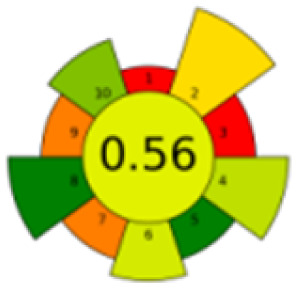	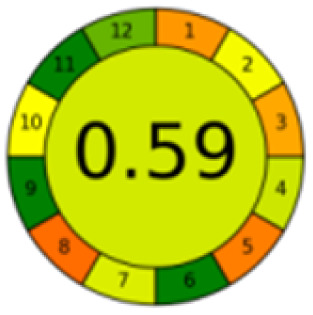
Serum^38^	SPE using Supelco LC-Ph cartridges	Clinical studies	0.025–5.00 μM	1.4 μM	4.45 μM	93.1–98.2	3.3	>10 min	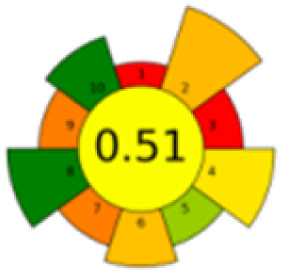	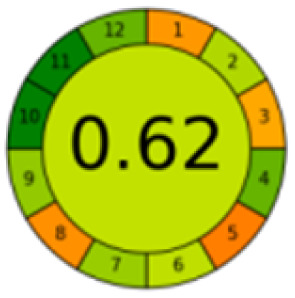
Serum^39^	Centrifugation	Clinical studies	26.3–631.2 μg/L	26 μg/L	9 μg/L	97.0	0	10 min	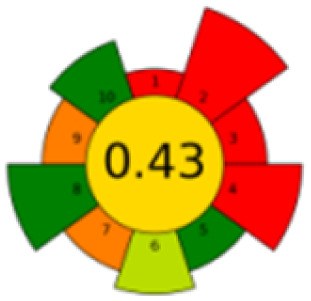	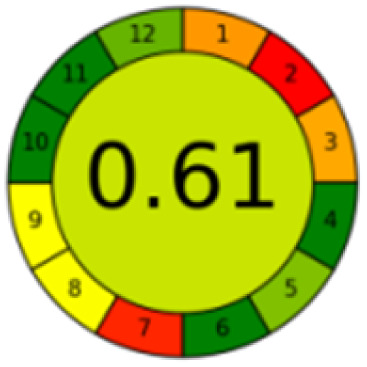
PBS,^40^ pH 7.4	Artificial permeability assay	Drugs for *in vitro* PAMPA assay	1.038–207.6 μM	0.03 μM	0.1 μM	—	0.45	>16 h	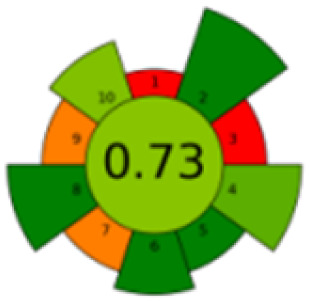	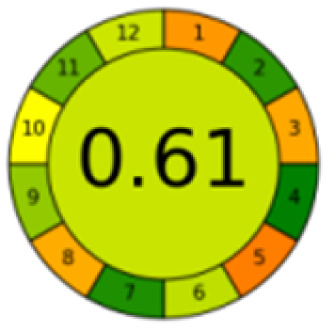

The comparison also presents an assessment of the reported methods on a recent assessment metric: AGREEprep and AGREE,^41^ which is based on Green Analytical Chemistry (GAC) principles. AGREE features a clock-shaped figure. The perimeter of the clock is divided into 12 sectors, each representing one GAC principle. AGREEprep is a metric tool for assessing the greenness of the sample preparation stage of an analytical procedure and AGREE is a tool for evaluating the environmental and occupational hazards, they all introduce a numerical evaluation for the assessed method in the core of its graph, ranging from (0.0–1.0). As demonstrated in Table 4, the proposed method has the greenest impact on both AGREEprep and AGREE with 0.78 score. Our method demonstrated a significant increase in environmental friendliness compared with traditional methods by utilizing minimal amounts of reagents and samples, low energy consumption, and highly efficient experimental procedures.

## 4 Conclusions

In summary, polystyrene (PS) nanofibres were coated with PEI, and the novel Pd(ii)/PEI/PS nanofibres resulting from coordination of PEI with Pd(ii) were produced. The physiochemical properties of the functionalised Pd(ii)/PEI/PS nanofibres were analysed using elemental analysis, EDX, BET surface area analysis, TGA and DTA, FT-IR, XPS, SEM, and TEM. The BET surface area provided information about the textural properties of the synthesised nanofibres, and EDX and XPS analysis confirmed a strong attachment of the metal complex to the PS/PEI support. TGA-DTA analysis was used to determine the thermal stabilities of the synthesised nanofibres, and SEM and TEM were employed to monitor their morphologies. The affinity of Pd with MTX and MTXPGs enables the newly prepared materials to effectively extract these highly polar pharmaceuticals from whole blood, urine and water samples. The Pd(ii)/PEI/PS nanofibres can be used to enrich and detect MTX concentrations as low as 0.025 μg/mL, which is below the lower limit of MTX concentrations that may pose a risk for toxicity to humans. This ensures effective and safe therapy for patients undergoing high-dose MTX treatments. Therefore, Pd(ii)/PEI/PS nanofibres could be used in monitoring these target compounds in both environmental and patient settings, thereby preventing excessive toxicity.

## Data availability

Data is available in main text.

## Author contributions

Conceptualization, X. J. K. and L. X.; resources, Q. S., X. H. Y. and W. H. G.; data analysis, J. Y. S., H. A. and L. X.; methodology, L. X. and X. J. K.; writing, reviewing, and editing, L. X.; supervision, X. J. K. All authors have read and agreed to the published version of the manuscript.

## Conflicts of interest

The authors declare that there is no conflict of interest.

## Supplementary Material

RA-015-D5RA00930H-s001
