# Supplementary material for: A new kind of polystyrene/polyethyleneimine nanofibres coordinated with palladium for fast and efficient extraction of methotrexate and its polyglutamate metabolites in different matrices
Source: RSC Adv. 2025 May 8;15(19):15017–28. doi: 10.1039/d5ra00930h (PMC12060134; doi:10.1039/d5ra00930h)
Supplement: RA-015-D5RA00930H-s001 [file RA-015-D5RA00930H-s001.pdf]

This journal is © The Royal Society of Chemistry 2025

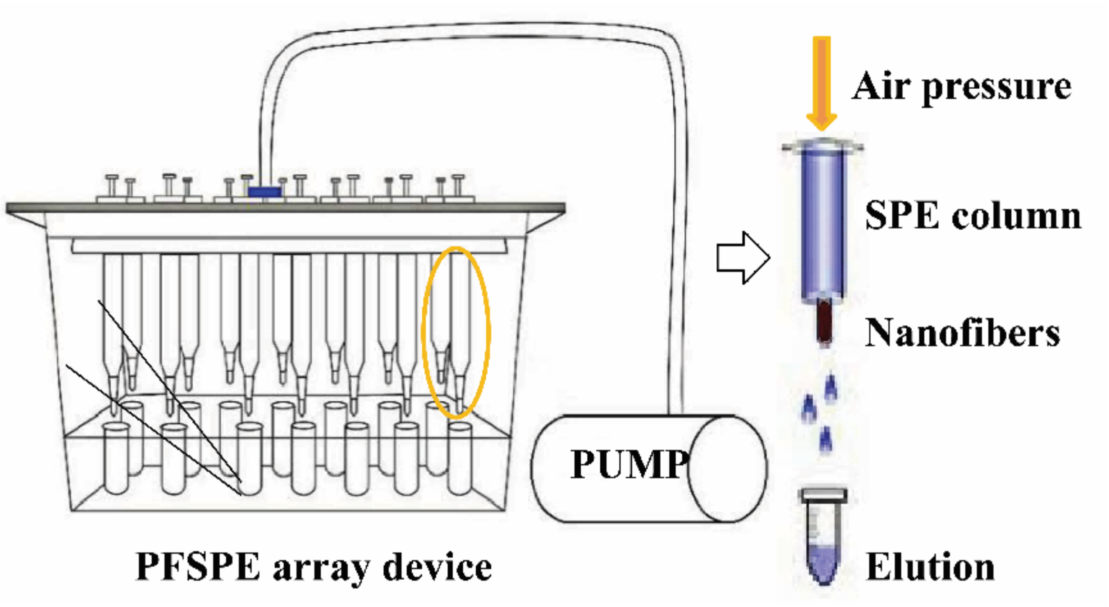

Supplementary Figure 1. Schematic diagram of an arrayed air-pumped SPE processor and SPE column.

FTIR spectroscopy

MTX molecule contains multiple electronegative elements, such as nitrogen and oxygen. Palladium readily coordinate with nitrogen, oxygen, sulfur, etc.-containing organic compounds, including amines, imines, Polypeptide etc.[1-3].

The structural characteristics of the PS nanofibres, PS nanofibres coated with $PdCl_2$, and Pd(II)/PEI/PS nanofibres before and after MTX adsorption, as well as the eluted fibres, were investigated using FTIR (Supplementary Figure 2 a-e). The peaks at 2922 and 3025 cm^{-1} correspond to the stretching vibration of C−H bonds, while the peaks at 1600, 1492, and 1452 cm^{-1} correspond to the stretching vibration of C=C bonds in the phenyl ring[4]. The FTIR spectra of the $PdCl_2$-coated PS nanofibres showed no difference compared to PS. However, Pd(II)/PEI/PS fiber (c) and the eluted Pd(II)/PEI/PS fibers after MTX adsorption (e) exhibited absorption bands at 1612 and 1635 cm^{-1} (highlighted in the red box), which can be attributed to the vibrations of the PEI-Pd bond in the coordination of metal ion[1]. The FTIR spectrum of the Pd(II)/PEI/PS nanofibres, the Pd(II)/PEI/PS nanofibres after MTX adsorption and elution, also exhibit absorption peaks of the N-H group at 3294 cm^{-1}, which indicate the successful modification of PEI and $PdCl_2$ onto the surface of the PS nanofibres[3]. However, after MTX adsorption on Pd(II)/PEI/PS fibres (d), the absorption bands of Pd(II)/PEI/PS were weakened, indicating the coordination of MTX with Pd on the Pd(II)/PEI/PS nanofibres.

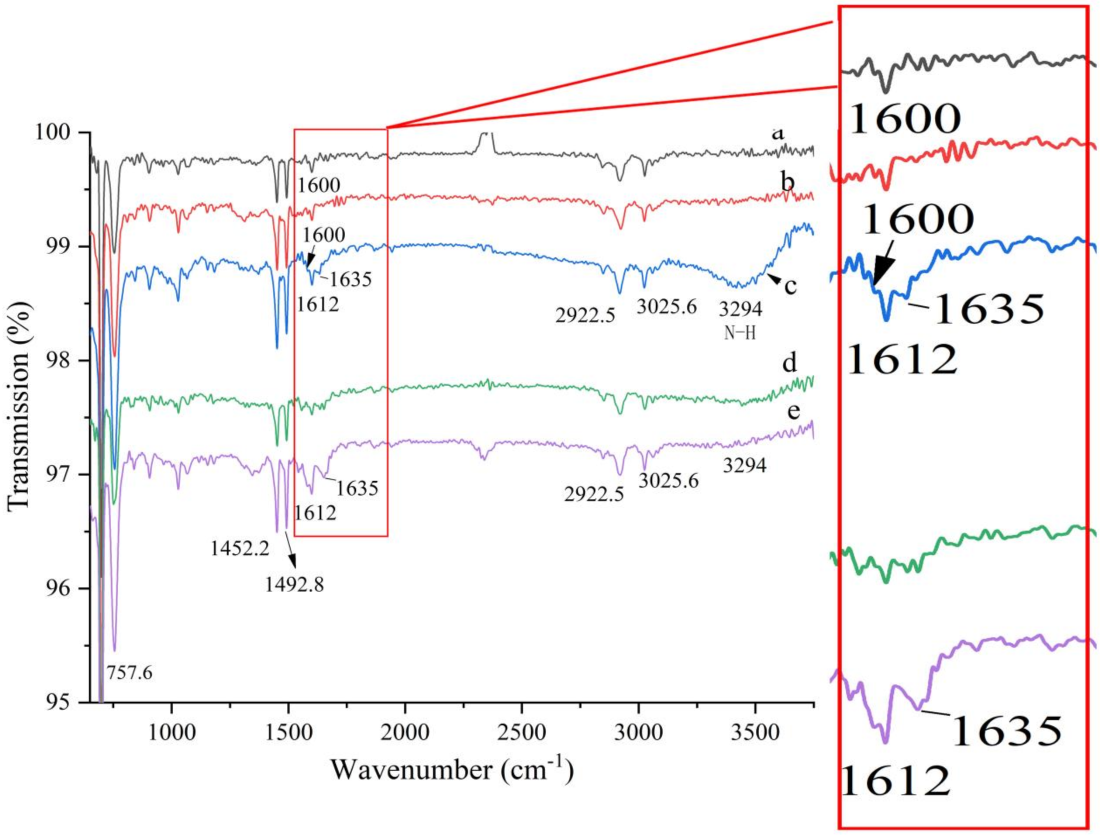

Supplementary Figure 2. FT-IR spectra of (a) PS nanofibres, (b) $PdCl_2$ coating PS nanofibres, (c) Pd(II)/PEI/PS nanofibres, (d) Pd(II)/PEI/PS nanofibres after adsorption of MTX, and (e) the MTX adsorbed Pd(II)/PEI/PS fibres after elution.

X-ray photoelectron spectra analysis

The compositions of the Pd-PS/PEI multilayers were analysed by the X-ray photoelectron spectroscopy, which revealed several peaks in the binding energy range from 0 to 1400 eV. As shown in Supplementary Figure. 3, three elements were detected, that is, C(1s), Pd ($3d_{3/2}$, $3d_{5/2}$), and N(1s), at the binding energies of 284.8, 337.8/343, and 399.9 eV, respectively. The C and N elements were from PEI, while Pd was from the connector of the PEI-Pd structure. These data indicate that Pd(II) ions were coordinated with PEI, which confirms the formation of Pd-PEI interactions.

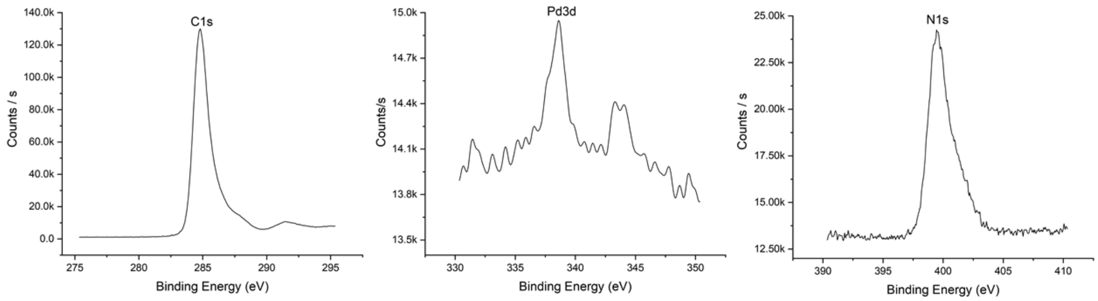

Supplementary Figure 3. XPS spectra for Pd(II)/PEI/PS nanofibres in the interaction with MTX.

Thermal analysis

Thermal analyses (TGA and DTA) of the (a) PS nanofibres, (b) PEI coated PS nanofibres, (c) Pd(II)/PEI/PS nanofibres before and after adsorption of MTX were carried out in air to investigate the thermal stabilities of the synthesised materials; the results are shown in Supplementary Figure. 4. TGA plots of all the synthesised and modified samples show approximately 5% weight loss below 100 °C caused by the desorption of physisorbed water molecules. The 60-90 weight% loss observed between 410 °C and 500 °C for all the nanofibres correspond to the matrix decomposition of the polystyrene framework (Supplementary Figure. 4A and B). PS-PEI nanofibres and Pd(II)/PEI/PS nanofibres exhibit broad peaks between 350 °C and 410 °C, which may be due to the removal of PEI layers from the nanofibres (Supplementary Figure. 4A(b, c) and B(b, c)). The broad peaks between 250 °C and 350 °C, which appeared only in the Pd(II)/PEI/PS nanofibres, may contribute to the decomposition of the -N-Pd(II) complex occluded on the surface (Supplementary Figure. 4A(c) and B(c)). The DTA plot of Pd(II)/PEI/PS nanofibres after adsorption of MTX (Supplementary Figure. 4B(d)) shows five distinct exothermic peaks between 225–275 °C, 275–310 °C, 310–350 °C, 375–425 °C, and 425–475 °C, which may due to the decomposition of the 1,2,3-triaozole Pd complex and -N-Pd(II) complex, PEI removal, the breakdown of the polystyrene framework and the decomposition of matrix benzene moieties.

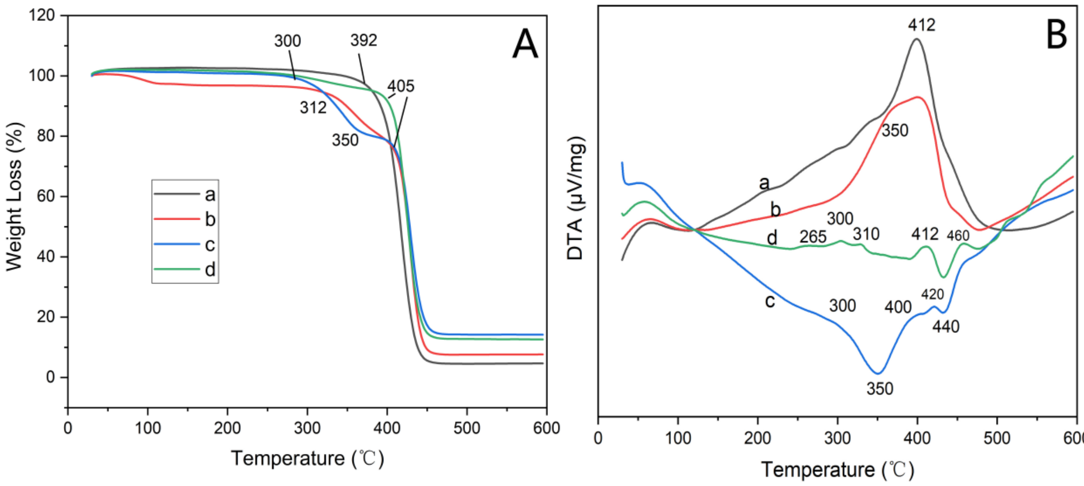

Supplementary Figure 4. (A) TGA and (B) DTA plots of (a) PS nanofibres, (b) PEI/PS nanofibres, (c) Pd(II)/PEI/PS nanofibres, and (d) Pd(II)/PEI/PS nanofibres after adsorption with MTX.

References

1. M. H. A. Drelinkiewicz, S. Quillard, C. Paluszkiewicz, *J. Mole. Struc.*, 1999, **511-512**, 205–215.
2. I. Lamego, M. P. Marques, I. F. Duarte, A. S. Martins, H. Oliveira and A. M. Gil, *J. Proteome. Res.*, 2017, **16**, 1773-1783.
3. J. M. Kozowski H, *Chem. Phys. Lett.*, 1977, **47**, 452-456.
4. Ž. D. Bugarčić, Jovana Bogojeski, and Rudi van Eldik. , *Coordina.Chem. Rev.*, 2015, **292**, 91-106.
